# Analysis of fluid flow around a beating artificial cilium

**DOI:** 10.3762/bjnano.3.16

**Published:** 2012-02-24

**Authors:** Mojca Vilfan, Gašper Kokot, Andrej Vilfan, Natan Osterman, Blaž Kavčič, Igor Poberaj, Dušan Babič

**Affiliations:** 1 J. Stefan Institute, Jamova 39, 1000 Ljubljana, Slovenia; 2 LPKF Laser & Elektronika d.o.o, Polica 33, 4202 Naklo, Slovenia; 3 Department of Physics, Jadranska 19, University of Ljubljana, 1000 Ljubljana, Slovenia

**Keywords:** biomimetics, fluid flow, low Reynolds number hydrodynamics, magneto-optical tweezers, microfluidics

## Abstract

Biological cilia are found on surfaces of some microorganisms and on surfaces of many eukaryotic cells where they interact with the surrounding fluid. The periodic beating of the cilia is asymmetric, resulting in directed swimming of unicellular organisms or in generation of a fluid flow above a ciliated surface in multicellular ones. Following the biological example, externally driven artificial cilia have recently been successfully implemented as micropumps and mixers. However, biomimetic systems are useful not only in microfluidic applications, but can also serve as model systems for the study of fundamental hydrodynamic phenomena in biological samples. To gain insight into the basic principles governing propulsion and fluid pumping on a micron level, we investigated hydrodynamics around one beating artificial cilium. The cilium was composed of superparamagnetic particles and driven along a tilted cone by a varying external magnetic field. Nonmagnetic tracer particles were used for monitoring the fluid flow generated by the cilium. The average flow velocity in the pumping direction was obtained as a function of different parameters, such as the rotation frequency, the asymmetry of the beat pattern, and the cilium length. We also calculated the velocity field around the beating cilium by using the analytical far-field expansion. The measured average flow velocity and the theoretical prediction show an excellent agreement.

## Introduction

The ability to move or to generate a flow in the surrounding medium is essential for living organisms. Unicellular organisms, for example, move when searching for food or better living conditions. In multicellular organisms, generation of a fluid flow above a surface is crucial for transporting an ovum in the Fallopian tubes, or for moving mucus in the respiratory tract, to name just two examples found in humans. Motion of fluid is also vital for embryonic development in vertebrates as directed flow establishes the left–right body asymmetry [1].

In all these cases, the fluid flow is generated by the same mechanism: By the beating of elongated hairlike protrusions, known as cilia, on the cell surfaces. Cilia are typically several micrometers long and only around 250 nm thick. They move periodically with the frequency of some tens of hertz, but their two-phase beat pattern is asymmetric and rather complex: During the effective stroke, the outstretched cilium propels the fluid like an oar, whereas during the recovery stroke the bent cilium returns to the initial position by sweeping along the surface in such a way that produces as little backward flow as possible [2].

The asymmetry of the ciliary beat pattern is a good illustration of Purcell’s theorem [3]. The theorem states that at low Reynolds numbers, where inertia is completely negligible and all motion is overdamped, directed swimming or pumping of fluid requires a nonreciprocal motion. If a cilium moved just back and forth along the same path, the resulting fluid flow would be averaged out. Cilia therefore beat asymmetrically as described above, bacterial flagella rotate as corkscrews and most eukaryotic flagella beat in a wavelike fashion.

In microfluidic applications, the small characteristic dimensions of the systems result in small Reynolds numbers, creating conditions comparable to the operating conditions of biological cilia. The efficiency of the ciliary pumping mechanism leads to the idea of using a similar principle when designing artificial cilia that would act as pumps and mixers in microfluidic devices. The artificial cilia would be periodically driven by an external force, for example by an electric or a magnetic field, and as long as it beats in an asymmetric manner, it should generate flow. Initial attempts to create externally driven artificial cilia resulted in nanorods manufactured from magnetic–polymeric composite materials [4]. The cilia were actuated in a simple periodic motion by a moving permanent magnet. Metal-coated polymer films have been used for the fabrication of electrostatically driven artificial cilia and used as mixers and pumps [5–6]. Light-driven microactuators have been manufactured using azo-doped liquid-crystal elastomers [7], although the speed of actuation that can be achieved with such a mechanism is presently too low for fluid pumping.

**Figure 1:**
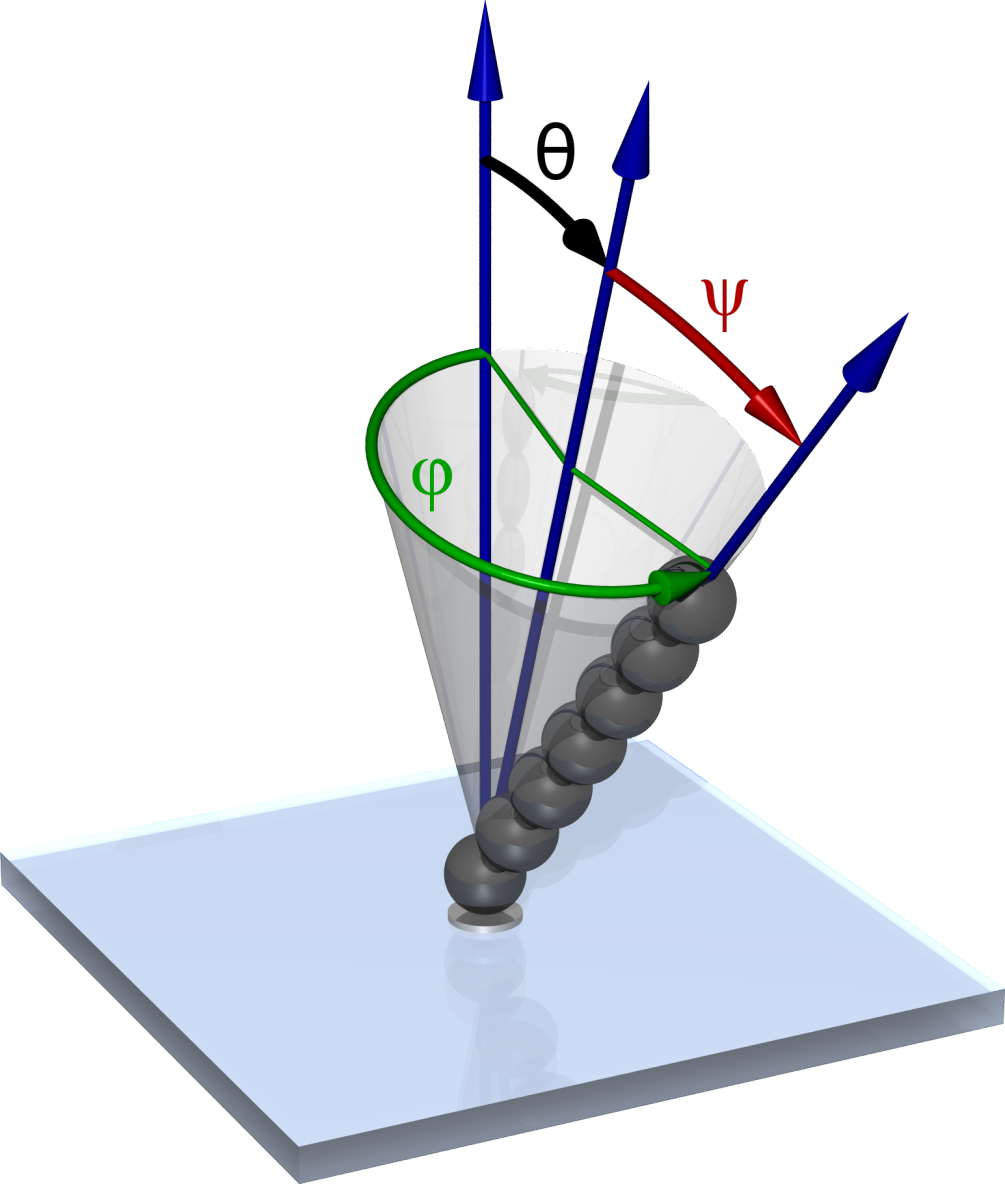
The artificial cilium is made of superparamagnetic beads. An external magnetic field is used to actuate the cilium in a periodic manner along a tilted inverted cone, defined by the tilt angle θ, semi-cone angle ψ and angular frequency 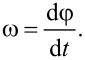

We recently successfully manufactured self-assembled artificial cilia driven by an external magnetic field and proved that their asymmetric beating generated a directed fluid flow [8–9]. The artificial cilia were formed as stable yet flexible chains of superparamagnetic colloidal particles and were driven along a tilted inverted cone (Figure 1). An array of such cilia pumped the surrounding fluid in one direction, and the velocity profile of the flow above a ciliated surface was measured [8]. At the same time, Sing et al. applied a similar principle to create chains of magnetic particles that were not anchored, but rather tumbled along the surface, which also has the potential for effecting fluid pumping [10]. Shields and co-workers fabricated a large array of flexible magnetic cilia and implemented a conical beat pattern [11]. They observed two sharply segregated regimes of fluid flow: Directed motion above the tips of the cilia, and mixing between the cilia. Coq et al. reported on investigations of the effect of the hydrodynamic interaction on the collective dynamics of large microcarpets [12]. Theoretical studies have also been performed in which different beat patterns were analysed, including planar [13–14] and conical motion [15–16]. The pumping performance of cilia can be enhanced by metachronal coordination [13,17], which has already been experimentally realised in a system of densely arranged cilia actuated by a rotating permanent magnet [18].

In this paper, we present a detailed analysis of the fluid flow around one beating artificial cilium. An analytical expression for the time-averaged flow velocity around one beating cilium is calculated for distances on the order of the cilium length. Different contributions are visualised and their effects on the pumping discussed. A comparison of the experimental results with the far-field expansion of the flow proves that higher-order terms need to be taken into account when calculating the fluid flow around a beating cilium.

## Results and Discussion

### Measuring the average flow velocity

The artificial cilium is composed of superparamagnetic particles held together by an external magnetic field. The magnetic field gives the cilium structural stability and at the same time enables controlled actuation describing the surface of a tilted cone. Fluid flow around the beating cilium was observed as a function of frequency, asymmetry (determined by the tilt angle), cilium length *L*, and height *z* above the surface, to which the cilium was attached. In order to observe the generated fluid flow, nonmagnetic tracer particles were introduced into the system and their motion was recorded.

**Figure 2:**
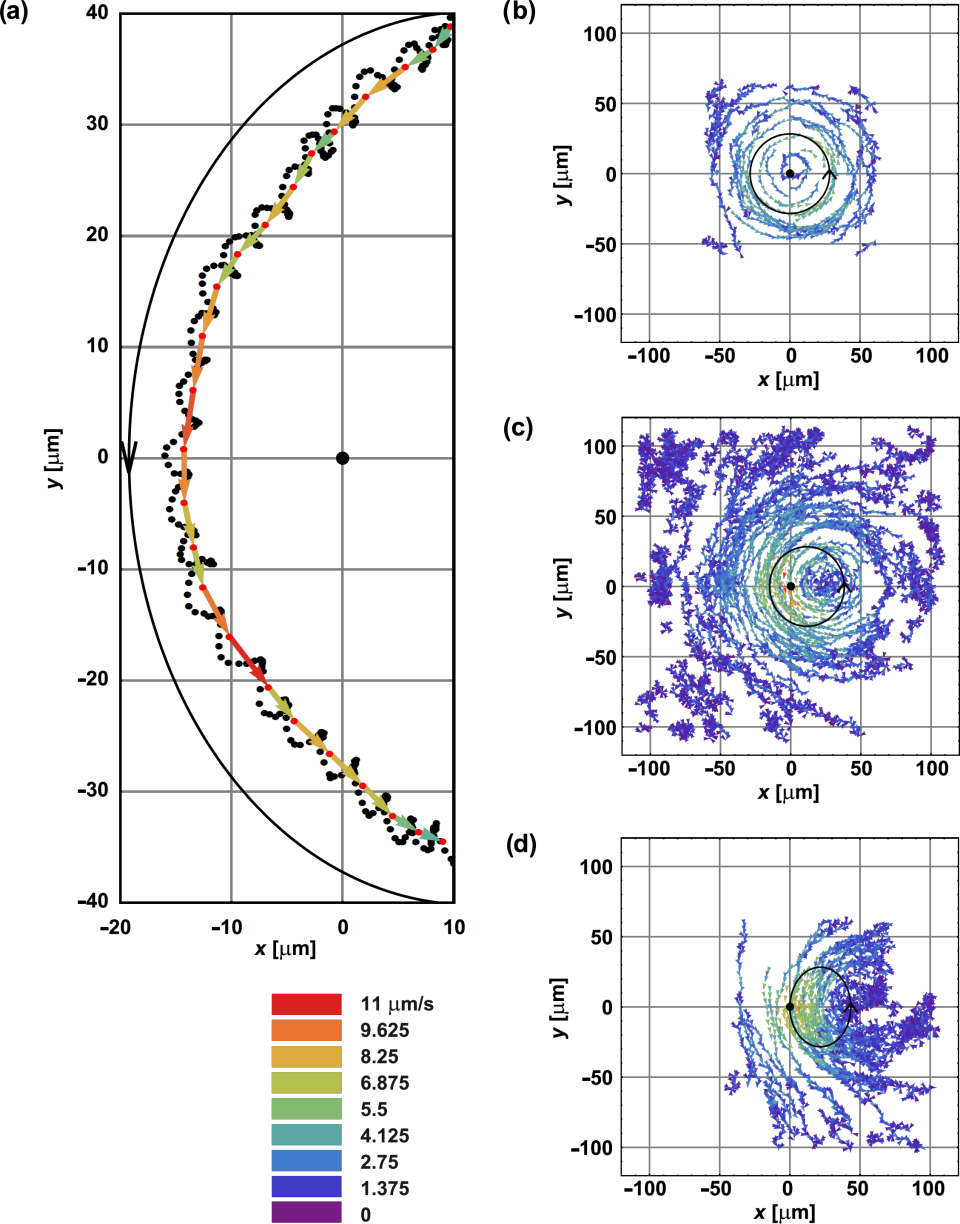
Fluid flow around a beating artificial cilium. The cilium was anchored to the surface at (0,0) and moved along an inverted cone. The calculated path of the tip of the cilium is shown as black closed curve. The flow around it was mapped by using nonmagnetic tracer particles. (a) The position of the tracer particle (black dots) was recorded every 30 ms. For each cilium cycle, the tracer positions were averaged and the time-averaged positions were obtained (red dots). The displacements between two consecutive red dots are shown as arrows. The colours of the arrows correspond to the velocities. With the rotational motion averaged out, a net translation of the particle is observed, indicating directed pumping of the surrounding fluid. Experimental parameters: Cilium length *L* = 44 μm, ψ = 40°, θ = 20°, rotating frequency ω/2π = 1 Hz, height above the surface *z* = 57.2 μm. (b)–(d) Combined traces of particles for three different cone tilt angles: the same data as in [9]. (b) θ = 0; (c) θ = 20°; and (d) θ = 40°. Other parameters are the same as in (a).

A typical trajectory of a tracer particle is shown in Figure 2a. The cilium was attached to the surface at (0,0) and the cone tilted in the +*x* direction. The motion of the cilium was anticlockwise, and the flow was generated in the −*y* direction. Black dots in Figure 2a are the actual positions of one tracer particle as recorded through an optical microscope, roughly 30 ms apart. One can see three distinct contributions to the motion of the tracer particle: The first is the circular motion with the periodicity matching the periodic motion of the cilium. For each cycle, the time-averaged position was calculated (red dots), which was later used to obtain the flow velocity, represented with the arrows. The colours of the arrows specify the velocity amplitude.

The second component of the trajectory is a translation in the −*y* direction that follows the generated directed fluid flow. Since only one cilium was used in the experiment, the translational flow is not straight but has an additional rotational component that bends the flow around the cilium. In a larger array of cilia this rotational contribution is in general absent, but remains visible at the boundaries [9]. In order to map the whole area around a beating cilium, a large number of such trajectories were recorded and collected in one figure for each set of parameters. The obtained data for three different cone tilt angles are shown in Figure 2b–d.

In accordance with Purcell’s theorem, there is no net fluid flow if there is no asymmetry in the system (Figure 2b). In this case, the cone along which the cilium rotates is not tilted, and only the vortical pattern is observed, centred at the anchoring point of the cilium. By averaging the measured velocities, one observes no net pumping and the position-averaged velocities in both the *x* and *y* directions are 0 μm/s within the experimental error. Keeping the semicone angle constant at ψ = 40° and increasing the tilt angle θ, an increase in pumping velocity is observed, as shown in Figure 2c and Figure 2d. In Figure 2c, a larger area was mapped. The motion of the more distant particles is governed by Brownian motion, as can be seen from the relatively small velocities of the tracer particles in arbitrary directions. Despite mapping a larger region, an equal number of tracer particles for the same sized region was included in the calculation of the average velocity of the flow.

**Figure 3:**
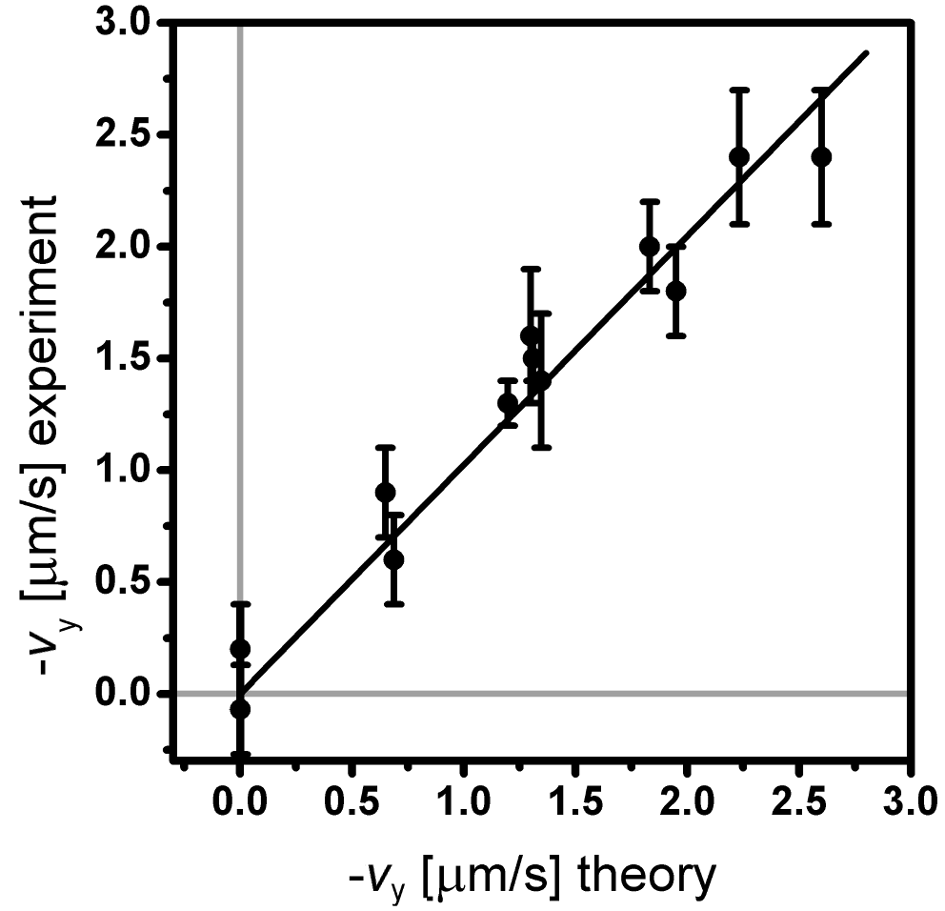
The time- and position-averaged flow velocities that were obtained for a variety of beating parameters are shown versus the theoretically calculated velocity (see data in Table 1). The solid line is a linear fit to the data with the obtained slope coefficient *k* = 1.02 ± 0.03, showing an excellent agreement between the theory and experiment.

The data was collected for a set of different parameters: Different tilt angles θ, different cone angles ψ, rotation frequency ω, height above the surface *z* and cilium length *L* [9]. All the time- and position-averaged pumping velocities combined are presented in Figure 3 and Table 1. They are compared to the theoretically obtained values, calculated for the same conditions and same parameters as used in the experiment.

**Table 1:** Experimental parameters, and theoretical and experimental values of the average velocity as shown in Figure 3.

*L* [μm]	θ [°]	ψ [°]	ω/2π [Hz]	*z* [μm]	−*v*_y_ [μm/s] (theor.)	−*v*_y_ [μm/s] (exp.)

44	0	40	1	57.2	0	−0.07 ± 0.2
30.8	0	40	1	40	0	0.2 ± 0.2
30.8	30.4	49.6	0.5	40	0.65	0.9 ± 0.2
30.8	20	40	1	40	0.69	0.6 ± 0.2
30.8	40	40	1	40	1.2	1.3 ± 0.1
30.8	30.4	49.6	1	40	1.3	1.6 ± 0.3
30.8	30.4	49.6	2	60	1.31	1.5 ± 0.1
44	20	40	1	57.2	1.35	1.4 ± 0.3
30.8	30.4	49.6	2	50	1.83	2.0 ± 0.2
30.8	30.4	49.6	1.5	40	1.95	1.8 ± 0.2
44	40	40	1	57.2	2.23	2.4 ± 0.3
30.8	30.4	49.6	2	40	2.6	2.4 ± 0.3

### Analytical calculation of the average flow velocity

A very basic approach to modelling the flows around a beating cilium, regardless of its precise beat pattern, consists of replacing the cilium with a small hypothetical particle circling along a tilted elliptical trajectory [19]. We have shown that the velocity field around the model cilium can be expanded in powers of the distance from the cilium anchoring point (*r*) and that the leading terms follow a 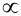 1/*r*^2^ dependence. The time-averaged flow velocity around a model cilium positioned at (0,0) for fluid pumping in the −*y* direction is

[1]
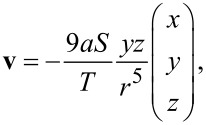


where *S* denotes the area of the elliptical trajectory projected onto the *y*–*z* plane, *a* the radius of the particle and *T* the beating period. The volume flow rate *Q* through the *x*–*z* plane is given by

[2]
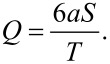


A somewhat more refined approach replaces the cilium with a rigid rod. This was used by Smith and co-workers [20], who combined the resistive-force theory to calculate the drag-force density along the cilium and the Blake tensor in the far-field approximation to calculate the volume fluid flow. They modelled the cilium as a slender rod of length *L* and radius *a*, moving along a cone with a semicone angle ψ, tilted by the angle θ from the vertical position. Their expression for the generated volume flow reads as

[3]
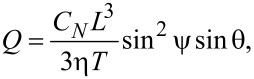


where *C_N_* is the transverse drag coefficient from the resistive force theory and η the water viscosity. Performing numerical simulations as described in [21] and comparing the calculated volume flow rate with Equation 3, we determined the effective coefficients for our system to be *C_N_*(*L*/2*a* = 7) = 1.29πη and *C_N_*(*L*/2*a* = 10) = 1.22πη.

The calculation can easily be refined by including the time-dependence of the flow velocity during a beat cycle. The time-dependent velocity is given by

[4]
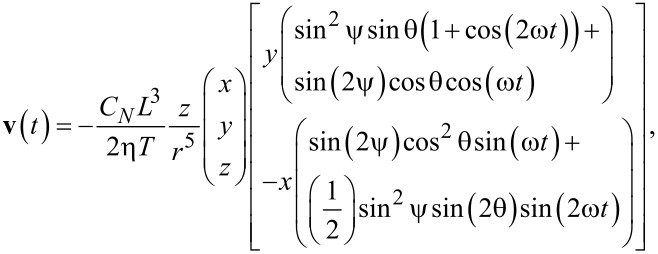


with ω = 2π/*T*.

One should note that this approach is very suitable for studying the interactions between beating cilia in the far-field approximation when |**x**| = *r* >> *L*. However, for distances |**x**| 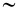 *L*, this simplified approach is not sufficient and higher-order terms have to be considered as well. From a series expansion of Blake’s tensor up to the order 1/*r*^3^, multiplied by the force density from the resistive-force theory (analogous to [20]), integrated over the length of a cilium and averaged over one period, we obtain the following expression for the average flow velocity:

[5]
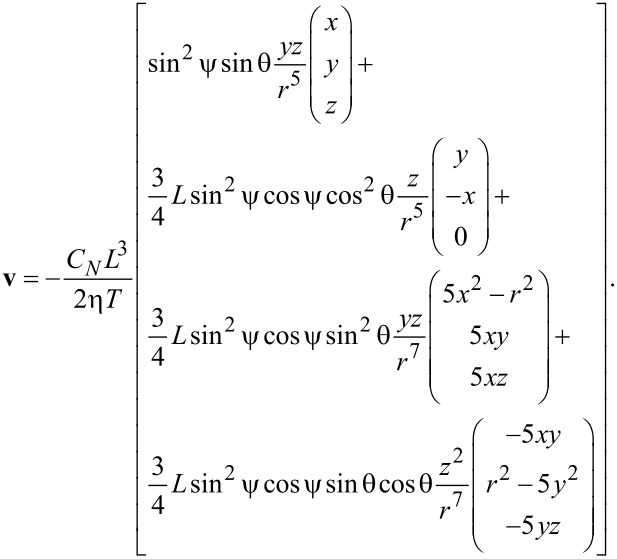


**Figure 4:**
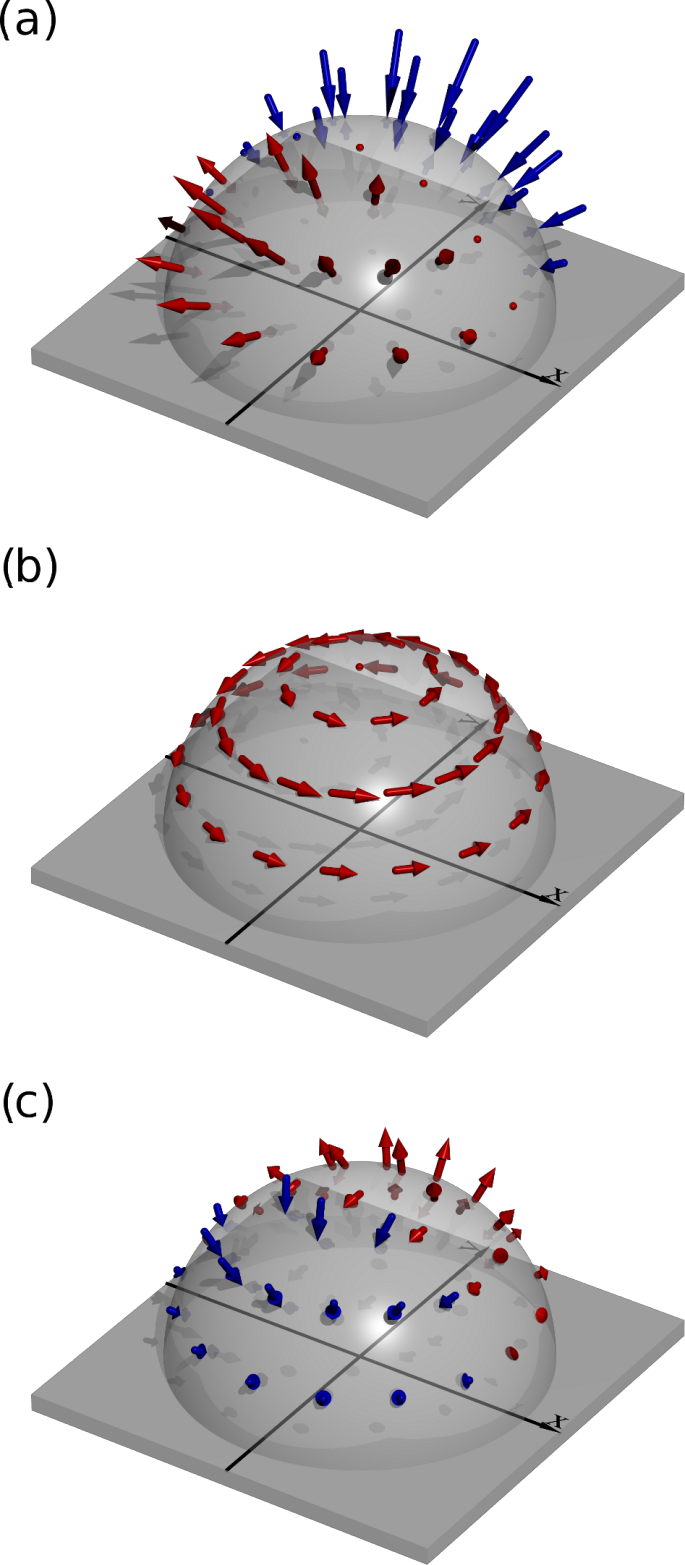
Calculated fluid flow around a beating cilium in the far-field approximation. Blue arrows indicate flow towards the cilium and red away from it. (a) The leading term in Equation 4 is the main contribution to the generated fluid flow. (b) The visualisation of the second term shows that this term contributes only to vortical motion. The second and the third term in Equation 4 do not contribute to the directed fluid flow as their contributions average out (third term is not shown). (c) Visualisation of the fourth term in Equation 4, which has to be taken into account when calculating the generated fluid flow.

The leading term exhibits the same dependence as in Equation 1. A graphical representation of this contribution to the flow velocity is shown in Figure 4a. The second term (Figure 4b) describes circular motion and the third term (not shown) is merely a shift of the first term in the *x* direction. The second and the third term do not contribute significantly to the flow in the *y* direction, as they both average out. The last term in Equation 4, however, does contribute to the averaged flow and has to be taken into account. Its graphical representation is shown in Figure 4c.

We can now calculate the average flow velocity for each set of parameters used in the experiment: *L*,*T*,ψ,θ and *z* and the corresponding *C_N_*(*L*). The calculated values are compared to the experimental ones in Table 1 and Figure 3. As the slope of the fitted line indicates, there is an excellent agreement between the experimental and the theoretical flow velocity.

## Conclusion

A number of microfluidic applications depend on efficient pumping and mixing of fluids in microscale channels [22–23]. We have previously shown that magnetically actuated artificial cilia successfully pump the surrounding fluid at low Reynolds numbers. Here we reported on detailed experimental and theoretical descriptions of fluid flow around one beating cilium. We mapped the flow around a beating cilium and calculated the average flow velocities for different beat parameters. A simplified theoretical far-field approximation was not sufficient to reproduce the experimental data. We took into account higher-order terms and the corrected theoretical result shows an excellent agreement with the measured data.

## Experimental

### Preparation of artificial cilium

We assembled the artificial cilium from monodisperse superparamagnetic beads (Dynabeads Epoxy M-450, Dynal Biotech, diameter 4.4 μm, standard deviation in bead diameter 55 nm [24]) in water. To prevent aggregation of the beads, we coated them with BSA (bovine serum albumin), 10 mg/mL, for 4 h in an ultrasonic bath. One end of the assembled chain was attached to the surface through prefabricated ferromagnetic-nickel anchoring sites. The nickel dots were manufactured by using a combination of photolithography and etching: First a 200 nm thick nickel layer was deposited on a microscope glass slide by a standard evaporation technique. A layer of negative photoresist (SU-8 2025, Microchem, adhesion promoter TI Prime, Microchemicals GmbH) was spin-coated onto the substrate. Direct illumination of the photoresist with an UV laser (Omikron Laserage GmbH, Bluephoton LDM375.20.CWA.L, 375 nm, Zeiss LD Plan-neofluar 10x/0.4 Korr objective) caused cross-linking of SU-8 molecules in the desired pattern [25]. The position of the laser beam was steered by acousto-optic deflectors (A.A. Opto-electronic, DTSXY-400-405) and a beam-steering controller (Aresis, d.o.o., BSC-160). After the photoresist was developed, the sample was ashed in post-glow oxygen plasma for 60 s and hard baked at 200 °C, leaving an SU-8 dot-array structure on nickel-covered glass. The slide was dipped in a cleaning solution (H_2_O/HNO_3_/HCl = 2:1:3) and etched with a standard chromium etchant (Sigma-Aldrich). The remaining photoresist was then removed with acetone and with delicate mechanical force, revealing the remaining nickel anchoring sites on the slide. The nickel dots were 5 μm in diameter and arranged in a square lattice with 28 μm between nearest neighbours. The glass slide with nickel dots was coated with BSA, 20 mg/mL, 5 h incubation, to prevent adhesion of the spheres onto the surface. A cell was made by gluing a cover slip onto the prepared slide with 200 μm spacers to ensure uniform sample thickness.

To monitor the fluid flow around a beating cilium, nonmagnetic tracer particles were introduced into the system. We used fluorescently labelled polystyrene spheres (Dragon Green, Bangs Laboratories, diameter 1 μm). Their concentration was approximately 6 × 10^−4^ particles per μm^3^, small enough for their influence on the flow to be negligible. We prepared a mixture of larger superparamagnetic and smaller nonmagnetic tracer beads in ultrapure water and filled the cell with the mixture by capillary action. The cell was later sealed with glue to prevent evaporation and possible currents. To avoid any wall effects, the flow was mapped and measured in the central part of the cell.

### Magneto-optical tweezers

**Figure 5:**
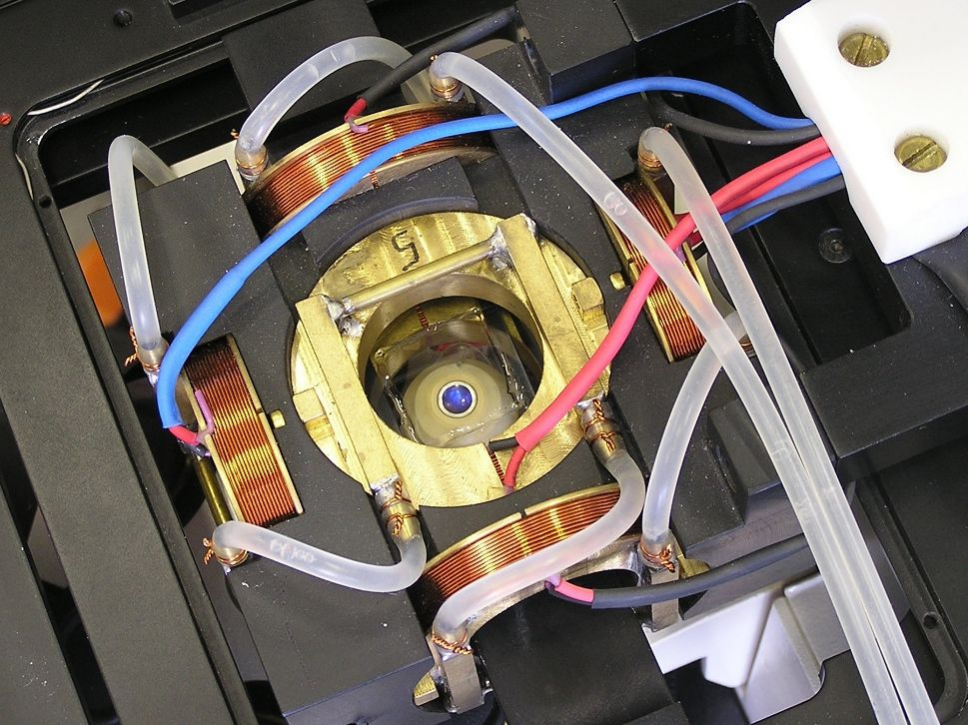
Magneto-optical tweezers used in the experiment. Three pairs of water-cooled coils ensured an almost homogeneous magnetic field at the sample.

Once the cell was filled with the bead mixture, the artificial cilium was assembled with optical tweezers that were built around an inverted optical microscope (Zeiss, Axiovert 200M, Achroplan 63/0.9W objective; Nd:YAG laser, 1064 nm, acousto-optic deflectors IntraAction and beam-steering controller Tweez by Aresis, d.o.o.). After the coarse initial positioning of the beads, the optical tweezers were switched off. The attractive force between the beads that stabilised the chain, the force between the chain and the anchoring site, and the actuation of the cilium were established with an external magnetic field. The optical tweezers were therefore equipped with a magnetic component that could generate a homogeneous magnetic field at the sample (Figure 5). Three orthogonal pairs of water-cooled coils were used to generate the magnetic field: All coils had a mean radius of 2.1 cm. The vertical pair with *n* = 216 turns was positioned 1.05 cm above and below the sample, whereas the horizontal pairs containing *n* = 243 turns were positioned 3.65 cm away from the centre of the sample. The magnetic field per unit current had a density of 10 mT/A in the vertical direction and 1.72 mT/A in both horizontal directions. The currents through the coils were individually regulated by a six-channel current source, which enabled generation of a nearly homogeneous magnetic field in an arbitrary direction and of varying magnitude. The typical magnetic-field density used in the experiments was between 5 and 7 mT, which is low enough to keep the magnetisation in the superparamagnetic particles well below the saturation value. The induced magnetic dipole moment in the beads was therefore proportional to the intensity of the magnetic field. This resulted in the formation of a long stable chain oriented parallel to the direction of the external magnetic field. When the direction of the magnetic field changed, the chain, that is the cilium, followed.

### Measuring the fluid flow velocity

The artificial cilium, which was driven with the external magnetic field in a periodic asymmetric manner, interacted with the surrounding fluid and generated a fluid flow. The flow was monitored by following the paths of the tracer particles with an electron-multiplying CCD camera (Hamamatsu Photonics, C9100-13) as shown in Figure 2. The motion within one beat cycle was time-averaged and a large number of trajectories were combined in one map. From these maps (three examples are shown in Figure 2b–d), the position-averaged flow velocity was calculated. Due to an uneven distribution of tracer particles, averaging the data over the whole sample was unreliable, and only the particles in the vicinity of the cilium were considered. We chose an area of four squares measuring 40 μm × 40 μm positioned at the centre of the projection of the cilium tip-sweep area. The issue of nonhomogeneous particle distribution was addressed in the following way: For each tracer particle that was randomly chosen from the first quadrant, three tracer particles at mirror sites in the remaining quadrants were selected, allowing a tolerance in position of 2.3 μm. When calculating the average flow velocity, 15 such quadruplets were taken into account and the procedure was repeated ten times to minimise the influence of the initial selection of the tracer particle. Although only pumping in the *y* direction was expected, we measured both *x* and *y* components of the velocity. The average velocity in *x* was indeed zero within the experimental error.

A similar procedure was implemented in order to obtain the theoretical values. The flow velocity in the *y* direction was calculated by integrating the time-averaged velocities over the same four squares at a given height *z*. All other parameters were the same as those in the experiment in order to achieve a reliable comparison.
